# Swallowing MRI—a reliable method for the evaluation of the postoperative gastroesophageal situs after Nissen fundoplication

**DOI:** 10.1007/s00330-018-5779-2

**Published:** 2018-11-12

**Authors:** Michael A. Arnoldner, Ivan Kristo, Matthias Paireder, Enrico P. Cosentini, Wolfgang Schima, Michael Weber, Sebastian F. Schoppmann, Christiane Kulinna-Cosentini

**Affiliations:** 1 Department of Biomedical Imaging and Image-Guided Therapy, Medical University of Vienna, Waehringer Guertel 18-20, 1090 Vienna, Austria; 2 Department of Radiology, Göttlicher Heiland Krankenhaus, Barmherzige Schwestern Krankenhaus, and Sankt Josef Krankenhaus, Vienna, Austria; 3 Department of Surgery, Medical University of Vienna, Vienna, Austria

**Keywords:** Magnetic resonance imaging, GERD, Fundoplication

## Abstract

**Purpose:**

To evaluate the diagnostic performance of swallowing MRI of the gastroesophageal junction (GEJ) in the postoperative care of patients after laparoscopic antireflux surgery (LARS)

**Material and methods:**

In this institutional review board-approved prospective study, 79 symptomatic patients (mean age, 52.3 years; range, 26–80 years) were evaluated after laparoscopic Nissen fundoplication. MRI findings were correlated with revision surgery, endoscopy, and high-resolution manometry (HRM) as standard of reference. MRI was performed on a 3.0-T unit using T2-weighted half-Fourier acquisition single-shot turbo spin echo (HASTE) sequences for anatomical assessment of the GEJ followed by dynamic MR swallowing (fast low-angle shot sequences). Four independent readers (two radiologists, two surgeons) rated 83 MR scans according to defined criteria, such as wrap disruption, slipping, recurrent hiatal hernia, and esophageal motility disorder.

**Results:**

Wrap disruption was correctly diagnosed concordantly with the standard of reference in 87.8%, slipping in 81.5%, and recurrent hiatal hernia in 84.9% of the cases. For esophageal motility disorder, MRI interpretation was consistent with manometry in 66.2% of the subjects. Interobserver analysis showed substantial agreement for recurrent hiatal hernia (*k* = 0.703), moderate agreement for wrap disruption (*k* = 0.585), and fair agreement for motility disorder and slipping (*k* = 0.234 and *k* = 0.200, respectively).

**Conclusion:**

MR swallowing readily depicts the major failure mechanisms of LARS and has good reliability even in non-experienced readers.

**Key Points:**

• *MR swallowing accurately readily depicts the major failure mechanisms of laparoscopic antireflux surgery and has good reliability even in non-experienced readers.*

*• It should be included in the preoperative workup for revision surgery after fundoplication.*

*• It will be of great benefit to surgeons in considering and planning a reoperation.*

## Introduction

Since its introduction in 1991, laparoscopic Nissen fundoplication has become the most common procedure in antireflux surgery [1] and long-term follow-up studies of 5 years and longer have shown a satisfactory rate of 93% [2]. However, 2–17% of these patients report new or recurrent postoperative symptoms, such as dysphagia, heartburn, and regurgitation [3]. The symptoms should resolve within 6 weeks; otherwise, a postsurgical failure as a possible reason for the symptoms has to be considered.

Postsurgical failure and its definition have been extensively discussed in the literature [4]. In some patients, failure means resumption of drug therapy. Others may benefit only from surgical revision [2], because the majority of cases are subject to morphological changes either attributable to the fundoplication wrap or the hiatal closure [5]. While different diagnostic follow-up options, such as manometry studies and endoscopy, exist, dynamic MR imaging was recently introduced as a promising non-invasive method for evaluating the gastroesophageal junction after Nissen fundoplication [6]. This method also may serve as an accessory method for the evaluation of esophageal motility [7, 8].

## Materials and methods

### Patient population

This prospective study was approved by the institutional review board, and written informed consent was obtained from each patient. The initial cohort included 79 patients (44 females and 35 males, 83 exams). Clinical inclusion criteria consisted of the following: prior laparoscopic antireflux surgery with Nissen fundoplication, recurrent or new clinical signs of gastroesophageal reflux disease (GERD), and/or other upper gastrointestinal tract symptoms such as gas bloating or dysphagia. Further general inclusion criteria comprised no contraindications for MRI and age over 18. Pregnant patients and patients who were unable to swallow in a supine position were excluded from our study. Patients were referred to our department after clinical evaluation by surgeons who specialized in upper gastrointestinal surgery. All patients were assessed at our institution.

Patients with new or recurrent symptoms were examined by endoscopy (*n* = 48), by high-resolution manometry (*n* = 53), and by an MR swallowing exam (*n* = 83). MR imaging was performed finally without knowledge of the results of the other examinations. The results of MR imaging were ultimately compared to the intraoperative results (*n* = 35) as the gold standard if the patients underwent reoperation. The decision for reoperation was made by the surgeon, depending on the individual clinical situation of the patient. The MR results of the patients who did not undergo reoperation were compared with the results of endoscopy and high-resolution manometry (HRM). In this group, endoscopy overruled HRM and was considered the standard of reference for all findings except esophageal motility disorder. Even though symptoms after fundoplication can be misleading, not all patients underwent HRM and endoscopy.

### MR imaging

MR imaging was performed on a 3-T MRI scanner (Magnetom Trio 3T, Siemens Healthineers) with a phased array coil placed upon the chest. Prior to the actual exam, the clinical history was obtained by one of the participating radiologists and the patient’s ability to swallow in the supine position was tested. The MRI protocol used in this study is shown in Table 1.

**Table 1:** MR scan parameters

Sequence	Voxel size (mm)	Slice thickness (mm)	Flip angle	TR	TE	Scan duration (min)
T2 HASTE axial	1.7 × 1.4 × 5.0	5	150°	1500	101	1:37
T2 HASTE coronal	2.0 × 1.6 × 3.0	3	150°	700	82	1:09
T2 HASTE sagittal	1.7 × 1.4 × 5.0	5	150°	1500	101	1:27
FLASH 3D coronal	3.1 × 1.6 × 7.0	7	8°	2.04	0.82	0:37
FLASH 3D sagittal	3.1 × 1.6 × 7.0	7	8°	2.04	0.82	0:37

A coronal T2w half-Fourier acquired single-shot turbo spin echo (HASTE) was obtained to depict the gastroesophageal junction (GEJ) and the entire course of the esophagus. To evaluate the position of the fundoplication wrap, T2w HASTE sequences were performed in the axial and sagittal planes. In addition, a paracoronal plane was obtained that followed the course of the lower esophagus.

In order to determine the optimum slice angle of the dynamic evaluation of bolus transit, a sagittal oblique T1w fast low-angle shot sequence (FLASH 3D) was centered on the lower esophagus in accordance with the T2w HASTE. The FLASH 3D sequence was then performed dynamically in the coronal and sagittal planes with three contiguous slices for better coverage of the esophagus and to compensate for plane displacement due to respiratory motion. To evaluate the motility of the esophagus, a cup filled with either a mixture of buttermilk and gadolinium-chelate (*n* = 46, gadoteric acid, Dotarem®, Guerbet) comprising 240 ml buttermilk and 6 ml gadoteric acid (dilution of 40:1) or Lumivision® (*n* = 37, dilution 1:1 Bendergruppe) was placed close to the patient’s head in the MR gantry. The patients were instructed to swallow a single gulp via a long plastic tube that was connected with the cup and then open their mouth in order to prevent repetitive swallowing.

MRI interpretation was performed on a PACS Workstation (IMPAX, Agfa-Gevaert), by two radiologists (R1 with 3 years of experience and R2 with 15 years of experience), as well as two surgeons (R3 and R4) with no active experience in MRI reading. However, the two surgeons were familiar with MR swallowing examinations from radiological-surgical conferences, which occur regularly. All readers were blinded to the examination results of other studies. Before reviewing the study images, the surgeons completed a short training session that included cases that were part of the study group. In addition, the radiologists performed a second reading session in consensus.

Diagnostic criteria for MRI were as follows:

1. Wrap location and integrity

The integrity of the fundoplication wrap was analyzed by the evaluation of a “pseudo-tumor-like appearance” at the GEJ. A wrap disruption was diagnosed when the “pseudo-tumor-like ring” could not be seen. A suspected partial tear, where parts of the presumed wrap still could be visualized, was also considered a disruption. Further, the GEJ was assessed for recurrent hiatal hernia and slipping. Slipping was defined as a telescope phenomenon, where a part of the stomach slipped through an intact fundoplication wrap. Hernia was diagnosed when gastric components could be visualized above the level of the hiatus. This also included wrap migration in the setting of an intact fundoplication wrap that was located above the diaphragm.

(2) Esophageal transit and reflux testing

An esophageal motility disorder was diagnosed when a delayed bolus transit was observed (transit time of a 10-ml liquid contrast medium bolus, which was swallowed in a single gulp, taking more than 20 s from upper to lower esophageal sphincters). In addition, non-propulsive contractions and missing esophageal clearance were considered suggestive of motility disorder. Additional sequences were acquired during a Valsalva maneuver to depict sliding hiatal hernia and recurrent reflux.

### Statistical analysis

All statistical analyses were performed using IBM SPSS Statistics for Windows Version 24.0 (IBM Corporation). Nominal data are presented using absolute frequencies and percentages. Metric data are presented using mean ± SD. In order to assess interrater agreement for all four readers, Fleiss kappa and their 95% confidence intervals (95% CI) were calculated. Kappa values of 0.81–1 were considered almost perfect, 0.61–0.8 substantial, 0.41–0.6 moderate, and 0.21–0.4 fair agreement.

## Results

All patients were able to complete the MR swallowing exam. The mean age at first presentation was 52.3 years (range, 26–80 years). The patients had undergone surgery from 6 weeks to 3 years before a swallowing MRI exam (mean, 2.6 years). The leading symptoms included dysphagia (*n* = 33), heartburn (*n* = 34), recurrent regurgitation (*n* = 17), and feeling of chest or upper abdominal pressure (*n* = 12).

No complications occurred during the examinations. Identification of all anatomic landmarks, such as the course of the esophagus, the esophagogastric junction, and the stomach, as well as the fundoplication region, was possible in all patients. The average examination time, including patient positioning on the MR table, was 32 ± 7 min.

### Revision surgery and non-surgical group

Revision surgery was performed in 35 of 79 patients (44.3%). In 10 cases (28.6%), a total wrap disruption was revealed. Two reports did not clarify the status of the fundoplication wrap. In the surgery group, 29 patients (82.9%) showed recurrent hiatal hernia, six of whom also showed slipping. Only one case showed slipping in the absence of hiatal hernia. The median time interval between primary and revision surgery was 3.4 years.

In the non-surgical group (*n* = 48), 28 endoscopic and 31 high-resolution manometry exams were suitable for correlation with imaging findings, resulting in 34 (70.1%) usable reports for wrap disruption, 33 (68.8%) for slipping, and 39 (81.3%) for recurrent hiatal hernia.

### Imaging findings compared to the standard of reference

The revision surgery group and the non-surgical group were separated for comparison, and consistencies for each reader are shown in Table 2. Overall consistencies (both groups) with the standard of reference were as follows:

**Table 2:** Per-case consistencies for each reader and for consensus reading

Abnormality			Reader 1	Reader 2	Reader 3	Reader 4	Consensus	Overall
Wrap disruption	Correctly identified		11/15 (73.4%)	14/15 (93.3%)	11/15 (73.4%)	13/15 (86.7%)	14/15 (93.3%)	87.8%
	Consistent with standard of reference	Surgery group	30/33 (90.9%)	27/33 (81.8%)	29/33 (87.9%)	30/33 (90.9%)	32/33 (97.0%)	
		Non-surgical group	28/34 (82.4%)	26/34 (76.5%)	30/34 (88.2%)	30/34 (88.2%)	32/34 (94.1%)	
Motility disorder	Correctly identified		9/21 (43.9%)	12/21 (57.1%)	9/21 (43.9%)	3/21 (14.3%)	11/21 (52.4%)	66.2%
	Consistent with HRM		32/53 (60.4%)	26/49 (53.1%)	38/52 (73.1%)	37/53 (69.8%)	39/53 (73.6%)	
Slipping	Correctly identified		4/11 (36.4%)	10/11 (90.9%)	3/11 (27.3%)	3/11 (27.3%)	10/11 (90.9%)	81.5%
	Consistent with standard of reference	Surgery group	21/33 (63.6%)	23/33 (69.7%)	27/33 (81.8%)	24/33 (72.7%)	29/33 (87.9%)	
		Non-surgical group	26/33 (78.8%)	28/33 (84.8%)	29/33 (87.9%)	30/33 (90.9%)	32/33 (97%)	
Recurrent hernia	Correctly identified		39/47 (82.3%)	41/47 (87.2%)	31/47 (66.0%)	35/47 (74.5%)	40/47 (87.2%)	84.9%
	Consistent with standard of reference	Surgery group	26/35 (74.3%)	29/35 (82.9%)	28/35 (80%)	29/35 (82.9%)	29/35 (82.9%)	
		Non-surgical group	32/39 (82.1%)	31/39 (79.5%)	37/39 (94.9%)	36/39 (92.3%)	37/39 (94.9%)	

#### Wrap disruption

**Fig. 1:**
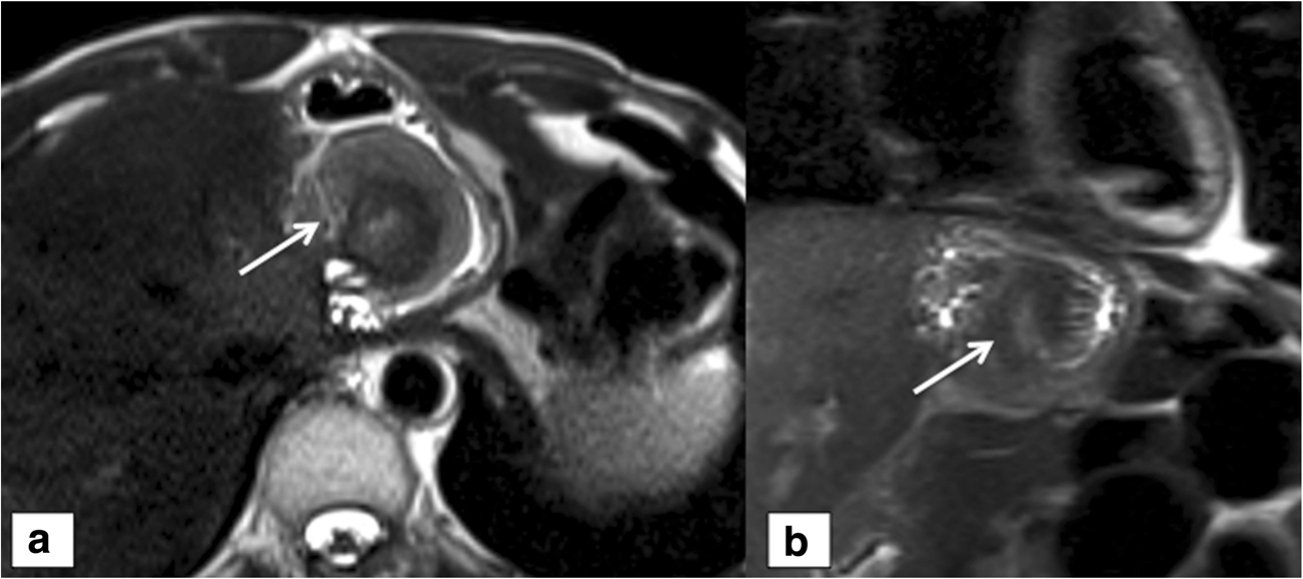
Normal wrap appearance. A 56-year-old male; axial (**a**) and coronal (**b**) T2 HASTE sequences show the typical “pseudo-tumor” appearance of an intact fundoplication wrap (arrows)

**Fig. 2:**
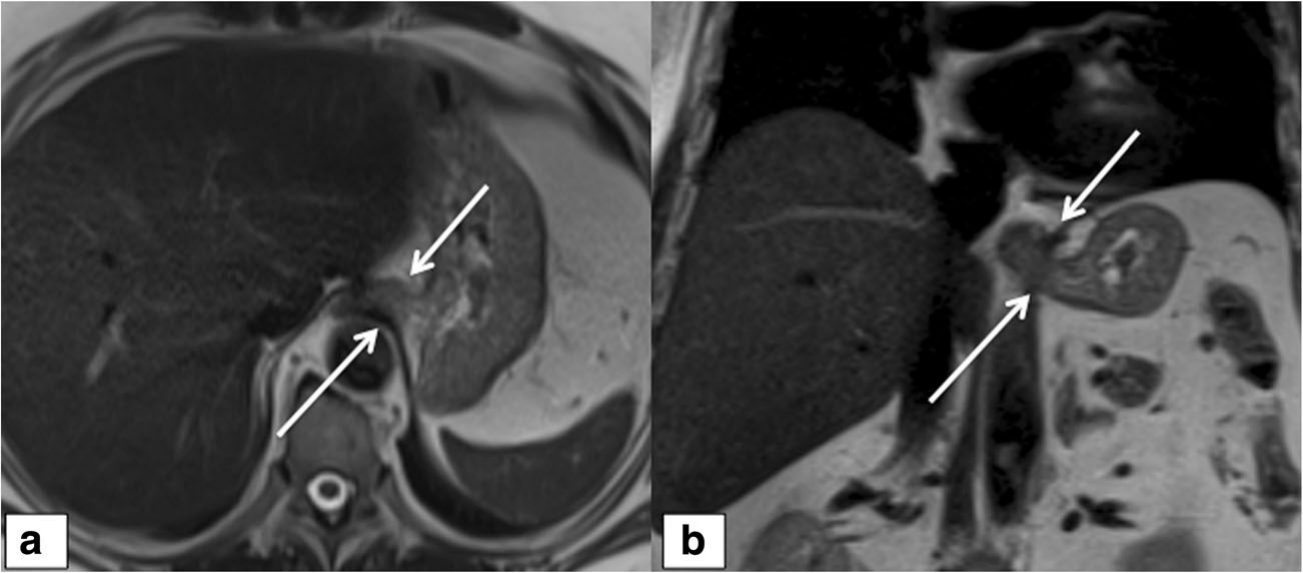
Wrap disruption. A 61-year-old male; axial (**a**) and coronal (**b**) T2 HASTE sequences showing complete wrap disruption. The typical “pseudo-tumor” is missed on the axial (**a**) and coronal (**b**) views (arrows)

In cases of wrap disruption (10 subjects in the surgery group and five in the non-surgical group), the “ring-like structure” (Fig. 1) could not be seen, as diagnosed in 14/15 (93.3%) patients (Fig. 2) by consensus; 9/15 (56.3%) cases were depicted correctly by all four readers. The radiologists diagnosed a wrap disruption in 11/15 (73.4%) (R1) and 14/15 (93.3%) (R2) cases, whereas the surgeons assessed the diagnosis of wrap disruption in 11/15 (73.4%) (R3) and 13/15 (86.7%) (R4) cases. Overall consistency was 87.8% (294/335); per-case consistencies with the percentage for each reader are shown in Table 2.

#### Slipping and recurrent hernia

**Fig. 3:**
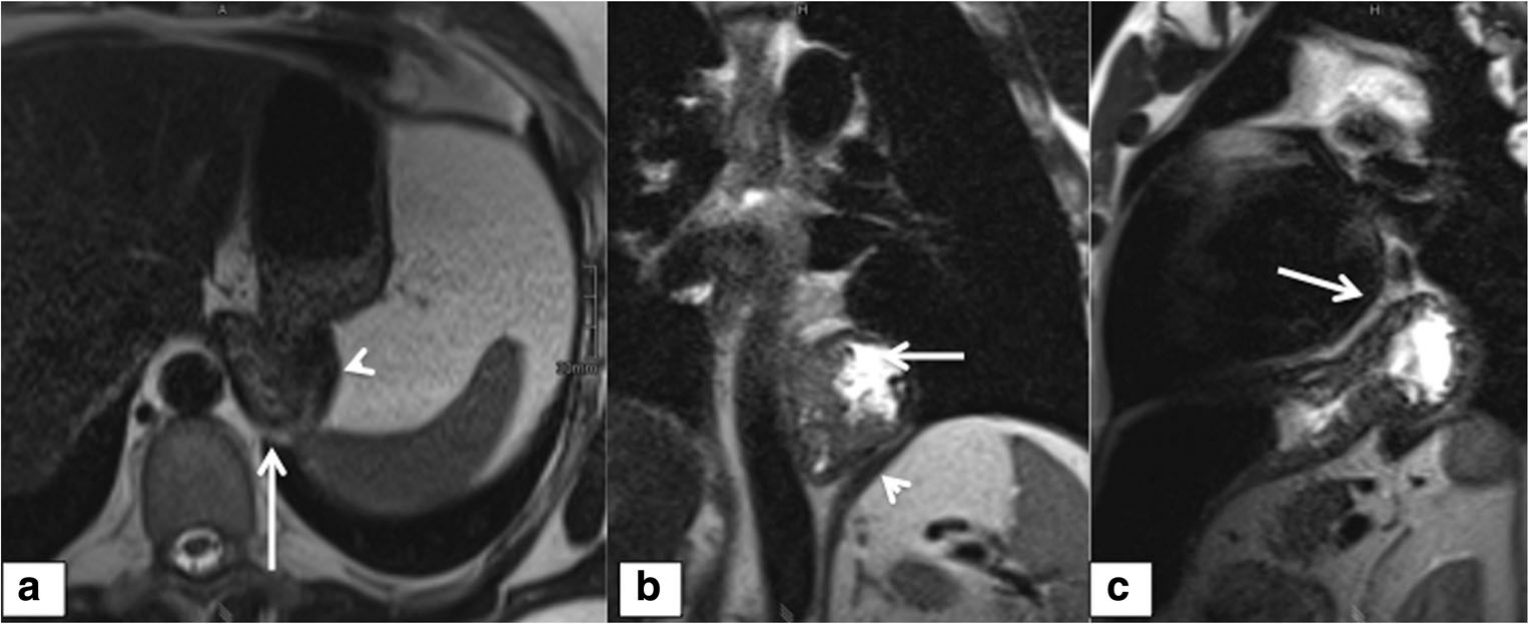
**a**–**c** Recurrent hernia. A 39-year-old male; axial, coronal, and sagittal T2 HASTE with parts of the stomach (arrow) located above the diaphragm (arrowhead)

**Fig. 4:**
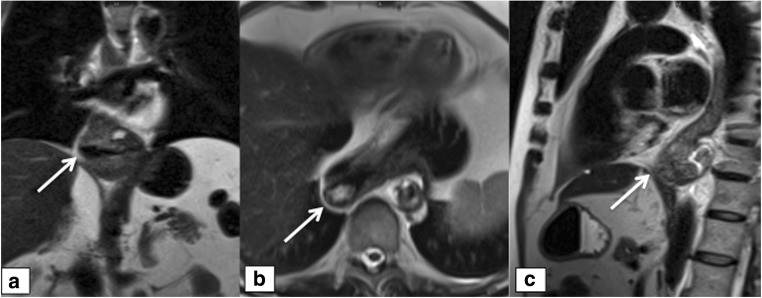
Wrap herniation. A 60-year-old male; T2 HASTE sequences in the coronal (**a**), axial (**b**), and sagittal (**c**) views show the entire wrap lies above the esophageal hiatus (arrows) with a concomitant recurrent hernia

Of the 29 patients (82.9%) with recurrent hiatal hernia (Fig. 3) in the surgery group, 24 (82.8%) were depicted by consensus. In the non-surgical group, 16/18 (88.9%) were detected by consensus, resulting in a total of 40/47 (85.1%) correctly identified recurrent hernias by consensus. Reader 1 detected a hiatal hernia in 39/47 (82.3%) cases and reader 2 detected 41/47 (87.2%) cases. The surgeons achieved a correct diagnosis in 31/47 (66.0%; R3) and 35/47 (74.5%; R4). Overall consistency was 84.9% (314/370). This was in contrast to a recurrent hernia, where parts of the stomach and sometimes the wrap had migrated above the hiatus (Fig. 4). Slipping was defined as parts of the stomach that had slipped through an intact wrap.

**Fig. 5:**
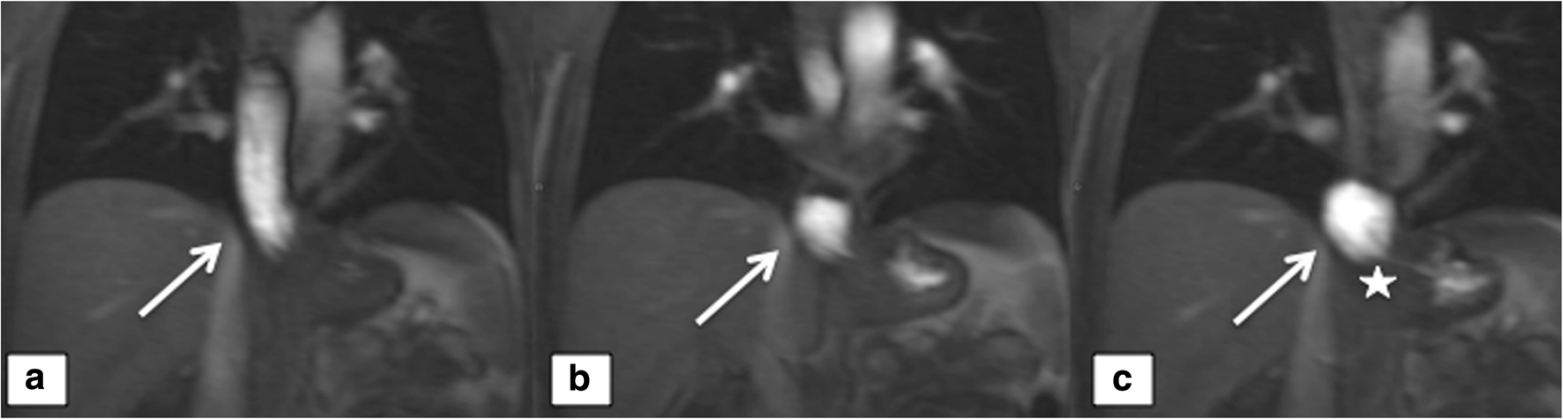
Slipping. A 49-year-old male; **a**–**c** dynamic, coronal T1w sequences during swallowing of a mixture of buttermilk and gadolinium-chelate show slipping of parts of the stomach (arrow on **b** and **c**). **a** shows the normal part of the distal esophagus (arrow on a) and **c** shows the intact wrap (asteriks) in the regular subdiphragmatic position

Slipping (Fig. 5) was found in a total of 11 subjects (seven cases in the surgery group) and correctly diagnosed in 10/11 patients (90.1%) by consensus. However, there was poor agreement, as no cases were detected correctly by all of the readers.

Overall consistency with the standard of reference for slipping proved to be 269/330 (81.5%); per-case consistencies for both slipping and recurrent hernia with the percentage for each reader are also shown in Table 2.

#### Motility disorder

High-resolution manometry reported esophageal motility disorders in 21 of 53 (39.6%) examined patients. There were 11/21 (52.4%) subjects with motility disorders who were also diagnosed on the MR swallowing exam by consensus, 9/21 (43.9%) (R1) and 12/21 (57.1%) (R2) by the radiologists, and 9/21 (43.9%) by R3 and 3/21 (14.3%) by R4. Overall consistency for motility disorders was 66.2% (172/260).

### Interrater agreement

All four readers rated 83 MRI exams for interobserver analysis. Table 3 shows the *k* values for overall and interrater agreement, as well as corresponding 95% confidence intervals. Overall, there was substantial agreement for recurrent hiatal hernia (*k* = 0.703) and moderate agreement for wrap disruption (*k* = 0.585). Motility disorders and slipping showed fair agreement (*k* = 0.234 and *k* = 0.200, respectively).

**Table 3:** Interrater agreement reflected by kappa values, with 95% confidence

Abnormality	Overall	CI	Surgeons	Radiologists
Wrap disruption	0.585	0.496–0.673	0.428	0.645
			75.9%	85.7%
Motility disorder	0.234	0.139–0.329	0.002	0.595
			59.8%	82.5%
Slipping	0.200	0.112–0.289	0.291	0.372
			81.9%	78.5%
Recurrent hernia	0.703	0.615–0.792	0.637	0.809

Degree of agreement: 0.81–1 = almost perfect, 0.61–0.8 = substantial, 0.41–0.6 = moderate, 0.21–0.4 = fair

*CI* confidence interval

Agreement was also evaluated between the surgeons and the radiologists, which showed lesser *k* values and a lesser degree of concordance for the surgeons (see also Table 2).

## Discussion

This study investigated the diagnostic performance and interrater agreement displayed by kappa statistics for MRI of the postoperative GEJ after laparoscopic Nissen fundoplication. MRI yielded good agreement with surgery and endoscopy for key morphologic findings that were causative for postoperative complaints. Interrater analysis showed moderate-to-substantial reliability. Furthermore, this study revealed that even less experienced and non-imaging-specialized readers achieve good detection of failure mechanisms. MR swallowing exams, therefore, provide a reliable non-invasive method for the assessment of complaints in patients after surgical treatment of GERD.

GERD is a mechanical disorder caused by a dysfunction of the lower esophageal sphincter (LES), failed esophageal peristalsis, or gastric emptying disorder. It is, therefore, a failure of the antireflux barrier that exposes the esophagus to gastric contents, with a broad spectrum of symptoms and complications that range from heartburn to malignant disease [9, 10]. Treatment options in long-term follow-up studies for up to 10.6 years [11–13] range from gastric acid suppression through proton pump inhibitors (PPI) to antireflux surgery. A recent study by Galmiche et al [14] reported that, with contemporary antireflux therapy for GERD, either by drug-induced acid suppression or by LARS, most patients remain in remission at 5 years. According to the guidelines of the Society of American Gastrointestinal and Endoscopic Surgeons, surgery should be offered to patients that (a) have failed medical treatment, (b) opt for surgery due to quality-of-life reasons, (c) have complications (e.g., Barrett’s esophagus), or (d) suffer from extra-esophageal manifestations of GERD (e.g., asthma) [15].

Although high satisfactory rates have been reported [2] in the literature, in up to 21% of the patients, fundoplication fails and recurrent symptoms, such as heartburn, dysphagia, or bloating, occur [3]. Those patients are subject to detailed multimodal examinations to elucidate the underlying cause. Furthermore, it has been shown that surgical success diminishes with each reoperation [4]. Compared with primary repair, revision surgery is associated with higher conversion rates to open surgery, longer operating times, and higher complication rates [15]. Thus, finding the reason for treatment failure is indispensable in the decision to reoperate or not and the planning of further treatment. This study supports the preliminary results of Kulinna-Cosentini et al [6], who showed that an MR swallowing exam offers a non-invasive method by which to visualize the postsurgical gastroesophageal situs and provides crucial anatomical information to the surgeon when considering a reoperation.

In comparison to fluoroscopy where the wrap can only be identified indirectly, MR imaging is the first radiological method that provides a direct view of the wrap itself. In the aforementioned preliminary study [6] with a small patient population, MR imaging yielded better results than endoscopy, which failed to diagnose the location of the wrap or even its disruption: Of the nine cases, five were misdiagnosed as recurrent hiatal hernia or newly acquired paraesophageal hernia, and, in four cases, there was a false diagnosis of reflux esophagitis. In the literature, endoscopy also shows a poor objective correlation with patient-reported symptoms, especially if the recurrent symptom is heartburn. Lord et al reported that no endoscopic abnormalities could be found in 62% of all symptomatic patients [16].

Although this study showed better results of MR imaging, imaging should not replace endoscopy. These methods are complementary in diagnosing the postoperative wrap situation.

As recurrent hernia is known to be the major cause of reoperation [4], it is not surprising that, in this study, 82.9% of the patients in the revision surgery group also had an hernia. An MR swallowing exam in consensus reading was correctly positive for hernia in 82.8% of cases, with five cases missed (17.2%). However, only 10.1% were missed in the non-surgical group. This might jeopardize surgery as the standard of reference, as results have been published, which indicate that high-resolution manometry is a reliable method for the detection of hernia. It has been hypothesized [17] that false-negative results on HRM may occur due to insufflation of gas during laparoscopy, thus exaggerating the number of hernias found at surgery.

For slipping and hernia, consensus terminology is important. According to the Hinder classification, there are four types of failure: disruption of the wrap (Hinder type I), stomach slippage above the diaphragm (Hinder type II), slipped Nissen (Hinder type III; the wrap slips down the stomach resulting in a telescope phenomenon with the gastric pouch above the level of the wrap), and transdiaphragmatic wrap herniation (Hinder type IV) [18]. In our institution, slipping is diagnosed if the stomach slips proximally through an intact fundoplication wrap (Hinder type III). However, recurrent hernia is diagnosed if there is displacement of gastric components above the level of the hiatus without regard to the status of the wrap (there can be both slipping and hernia). However, we are aware that various definitions of a “slipped Nissen” exist, including the transthoracic migration of the wrap [19], which is actually consistent with a Hinder type IV failure.

Slipping was found in a total of 11 subjects (seven cases in the surgery group) and correctly diagnosed in 10/11 patients (90.1%) by consensus. However, there was poor agreement, as no cases were detected correctly by all of the readers.

One explanation might be that a tube-like, malformed part of the gastric fundus was misinterpreted as the distal esophagus. Mostly, the so-called telescope phenomenon, or slipping, develops gradually if the cuff has been located too low and the intraabdominal part of the esophagus is not well mobilized during the first operation [20].

Wrap disruption has been reported in the literature in up to 12.8% of cases that undergo revision surgery [21]. In our study, reoperation revealed disruption in 28.6% of the cases. A correct diagnosis of wrap disruption was achieved by the more experienced reader (R2) of the radiologists and by R4 of the surgeons (93.3 and 86.7%, respectively), whereas R1 and R3 achieved a correct diagnosis in 73.4%.

A certain level of experience in reading MR swallowing seems to be important in the assessment of wrap disruption. Often, it can be difficult to differentiate between complete disruption and partial disruption, but clinical consequences in symptomatic patients are the main reason for reoperation. The LARS procedure performed in this study was Nissen fundoplication, where the gastric fundus is wrapped in a 360° fashion around the GEJ [22], resulting in a typical pseudo-ring-like appearance on MRI [6]. However, several variations of fundoplication exist (e.g., Toupet 270°) [23], which change the “normal” wrap presentation on MRI. Thus, it is crucial that the radiologist be familiar with the type of LARS performed to prevent reporting of a false-positive wrap disruption.

With a restored reflux barrier after fundoplication, dysphagia occurs in 3 to 17% [15]. Twenty-one of our patients (26.6%) were diagnosed with esophageal motility disorder on HRM, and an MR swallowing exam in consensus reading was able to correctly identify approximately half the cases (52.4%). However, there was only fair interrater agreement among the four readers, with overall *k* values of 0.200. HRM remains the absolute gold standard for motility disorders, but an MR swallowing exam may be able to identify causative morphologic changes, such as a too-tight wrap [6].

It is known that the main limitation of an MR swallowing exam is its low temporal resolution of two pictures/second, in contrast to videofluoroscopy with 25 frames/second [7, 24]. In accordance with the previous findings of Kulinna-Cosentini et al, where the sagittal oblique plane demonstrated the best visualization of the esophagus with a single-slice mean length of 16 cm on an MR swallowing exam [25], this also applied in our study.

This study has some limitations: Our main objective was to determine the interrater reliability. Since all diagnostic criteria were dichotomous variables (yes/no), agreement by chance in some cases is very likely. Otherwise, kappa statistics reflect chance-corrected agreement, which, on the other hand, may be excessive in dichotomous variables, resulting in too-low values. Second, in order to facilitate the reading, we did not make a distinction between partial or complete wrap disruption. However, it is very likely that symptomatic patients with suspected disruption—both partial and complete—will undergo revision surgery. Furthermore, it should be noted that swallowing a bolus of buttermilk consistency in the supine position does not simulate daily food intake and may cause false-positive findings in motility assessment. Moreover, it has been shown that including multiple water swallows and a solid meal test increase the diagnostic yield for HRM [26].

## Conclusion

MR swallowing exams readily depict the major failure mechanisms of LARS and provide good reliability, even in non-experienced readers. It will be of great benefit to surgeons in considering and planning a reoperation and should be included in the preoperative workup for revision surgery after fundoplication.

## Abbreviations

FLASH: Fast low-angle shot
GEJ: Gastroesophageal junction
GERD: Gastroesophageal reflux disease
HASTE: Half-Fourier acquisition single-shot turbo spin echo
HRM: High-resolution manometry
LARS: Laparoscopic antireflux surgery
LES: Lower esophageal sphincter
